# OSCC in Never-Smokers and Never-Drinkers Is Associated with Increased Expression of Tumor-Infiltrating Lymphocytes and Better Survival

**DOI:** 10.3390/cancers15102688

**Published:** 2023-05-10

**Authors:** Mathias Fiedler, Alisa Off, Jonas Eichberger, Steffen Spoerl, Johannes G. Schuderer, Juergen Taxis, Richard J. Bauer, Stephan Schreml, Torsten E. Reichert, Tobias Ettl, Florian Weber

**Affiliations:** 1 Department of Cranio- and Maxillofacial Surgery, Hospital of the University of Regensburg, Franz-Josef-Strauß-Allee 11, 93053 Regensburg, Germany; alisa.off@web.de (A.O.); jonas.eichberger@stud.uni-regensburg.de (J.E.); steffen.spoerl@ukr.de (S.S.); johannes.schuderer@ukr.de (J.G.S.); juergen.taxis@ukr.de (J.T.); torsten.reichert@ukr.de (T.E.R.); tobias.ettl@ukr.de (T.E.); 2 Insitute of Pathology, University of Regensburg, Franz-Josef-Strauß-Allee 11, 93053 Regensburg, Germany; florian.weber@ukr.de; 3 Department of Cranio- and Maxillofacial Surgery, Center for Medical Biotechnology, Hospital of the University of Regensburg, Franz-Josef-Strauß-Allee 11, 93053 Regensburg, Germany; 4 Department of Dermatology, Hospital of the University of Regensburg, Franz-Josef-Strauß-Allee 11, 93053 Regensburg, Germany; stephan.schreml@ukr.de

**Keywords:** oral squamous cell carcinoma, never-smoker, never-drinker, tumor-infiltrating lymphocytes, CD4, CD8, FoxP3, CD1a

## Abstract

**Simple Summary:**

A significant proportion of patients with oral squamous cell carcinoma do not have the typical risk factors of smoking and alcohol consumption in their medical history. Clinical and immunohistochemical studies have shown that patients with oral squamous cell carcinoma who have never smoked or drunk alcohol are more likely to be female and older at the time of diagnosis. In addition, this type of patient has a more frequent history of oral potentially malignant disorders and a better prognosis. There are also differences in the site of origin of the carcinoma between never-smokers/never-drinkers and smokers/drinkers. In addition, we were able to show that oral squamous cell carcinoma in never-smokers/never-drinkers has an independent immunologic microenvironment. These results may help to improve the treatment of oral squamous cell carcinoma in never-smokers/never-drinkers.

**Abstract:**

The aim of this study was to investigate the clinical, histopathologic, and immunologic differences of oral squamous cell carcinoma of never-smokers/never-drinkers and smokers/drinkers. Immunohistochemical staining for CD4, CD8, FoxP3, CD1a, and p16 was performed in 131 oral squamous cell carcinomas from smokers/drinkers and never-smokers/never-drinkers. Associations of smoking/drinking status with clinicopathologic data, immunohistochemical antibody expression, and survival were examined. Oral squamous cell carcinoma in never-smokers/never-drinkers was associated with the female gender (*p* < 0.001). Never-smokers/never-drinkers were older at diagnosis than smokers/drinkers (*p* < 0.001). Never-smokers/never-drinkers had more tumors in the maxilla, mandible, and tongue (*p* < 0.001). Pre-existing oral potentially malignant disorders appeared to be more common in never-smokers/never-drinkers (*p* < 0.001). Perineural invasion was more common in smokers/drinkers (*p* = 0.039). Never-smoking/never-drinking was associated with better overall survival (*p* = 0.004) and disease-specific survival (*p* = 0.029). High CD4+ T cell infiltration was associated with never-smoking/never-drinking (*p* = 0.008). Never-smokers/never-drinkers also showed increased CD8+ T cell infiltration (*p* = 0.001) and increased FoxP3+ Treg infiltration (*p* = 0.023). Furthermore, the total group of tumor-infiltrating lymphocytes was associated with never smoking/never drinking (*p* = 0.005). To conclude oral squamous cell carcinoma of the never-smokers/never-drinkers appears to be a distinct type of tumor, as it appears to have unique clinical and pathologic features and a more immunogenic microenvironment.

## 1. Introduction

Head and neck squamous cell carcinoma (HNSCC) is one of the most common tumors worldwide, with nearly 900,000 cases per year [1]. The majority of HNSCC arises from the oral cavity (oral squamous cell carcinoma = OSCC) [1]. Men are typically four times more likely to be affected by this tumor entity than women [1]. Similar to other aerodigestive tract tumors, the main risk factors are tobacco (both smoked and ‘smokeless’) and alcohol use [1]. Tobacco smoke contains a complex mixture of chemicals thought to cause cancer via DNA damage, when misreplicated, leads to an increased burden of somatic mutations and thus an increased likelihood of acquiring “driver” mutations in cancer genes [2]. DNA damage caused by acetaldehyde, which is produced during the breakdown of ethanol, is a known mechanism of alcohol carcinogenicity [3]. Furthermore, alcoholic drinks might act as a solvent to penetrate other carcinogens through the mucosa [3]. This is also a possible reason for the known synergistic effect of alcohol and tobacco consumption on the development of OSCC. Heavy alcohol consumption and concurrent tobacco use can increase the risk of developing OSCC by a factor of 35 [4]. However, about 10–15% of OSCC patients have never smoked or consumed alcohol outside the recommended limits [5]. Preliminary studies suggest that this group of OSCCs in NSND patients is a distinct subset of tumors [5,6]. For example, Koo et al. showed that this type of carcinoma occurs more frequently in women than men and is associated with better outcomes [6]. In addition, carcinomas in NSND patients appear to be localized in different areas of the oral cavity compared to patients who smoked and drank (SD) [6].

Cigarette smoke is known to be a modulator and promoter of chronic inflammation through a variety of mechanisms [7]. There is considerable evidence that alcohol consumption may affect the innate and adaptive immune system [8]. Therefore, differences in the immunologic microenvironment between NSND and SD OSCCs would not be surprising [5]. Tumor-infiltrating lymphocytes (TILs) are an important part of the tumor immunologic microenvironment. TILs include T lymphocytes such as CD4+ T-helper 1 (Th1) and T-helper 2 (Th2) cells as well as CD8+ cytotoxic T cells [9,10]. It is known that CD4+ T helper cells and CD8+ cytotoxic T cells protect against tumor growth and that a coordinated and balanced interplay between these subsets is required to protect the host from a developing tumor [10]. In addition to CD4+ T helper cells, the group of CD4+ T cells includes a subset of Forkhead box P3+ (FoxP3+) T regulatory lymphocytes (Tregs) [10]. Tregs are a kind of counterpart to the above cells as they play a key role in the process of tumor immune escape by producing the immunosuppressive cytokines IL-10 and TGF-β and consuming IL-2 [10]. In addition to TILs, various cells of the innate immune system, such as macrophages, CD1a+ dendritic cells (DCs), and natural-killer (NK) cells, are part of the tumor microenvironment [11]. Tumor-associated macrophages promote invasion, metastasis, angiogenesis, and immunosuppression of OSCC by synthesizing and releasing a variety of growth factors, cytokines, chemokines, and proteolytic enzymes [11]. DCs play one of the most important roles in the antitumor immune response, as it begins with the uptake of tumor-associated antigens by this cell type [10,12]. A more detailed investigation of possible differences in the immunological microenvironment in SD and NSND, and a more detailed description of these differences in NSND patients may help to improve the therapy options of this population.

The objective of this study was to investigate the clinical, histopathologic, and immunologic differences of OSCC of NSND and SD patients. For this purpose, we performed in a collective of OSCC a clinical data collection as well as immunohistochemical staining of some of the most important tumor-infiltrating immune cells, i.e., CD4+, CD8+, and FoxP3+ T-cells as well as CD1a+ DCs.

## 2. Materials and Methods

### 2.1. Patient Data Collection

The study cohort consisted of 131 patients with OSCC of various localizations. The study patients were diagnosed and staged between 2008 and 2021 at the Department of Oral and Maxillofacial Surgery, University Hospital Regensburg (Regensburg, Germany). Clinical and histopathologic data were collected from the patient’s medical records. Follow-up data were obtained from the Regensburg Tumor Registry. Never smokers were patients who never used tobacco of any kind. Never drinkers are patients who, on average, did not exceed the limit of 20 g per day. This study was approved by the local ethics committee (No. 22-2798-101). This study was also in accordance with the ethical standards of the Helsinki Declaration of 1964 and its subsequent amendments.

### 2.2. Immunohistochemistry (IHC)

#### 2.2.1. Preparation and Staining of Samples

Formalin-fixed, paraffin-embedded tumor tissue was available for all patients. The corresponding samples were obtained from the archive of the Institute of Pathology at the University of Regensburg, and tissue microarrays (TMA) were prepared according to the usual protocol [13]. Three 1.5 mm punches were taken from each tissue block at different representative sites. The punches were transferred to the TMA block, and 3 µm thick slices were cut from each TMA and mounted onto SuperFrost^®^ Plus Microscope Slides (R. Langenbrinck Labor- u. Medizintechnik, Emmendingen, Germany). Subsequent immunohistochemical staining was performed according to the standard protocol of the Institute of Pathology, University of Regensburg, with a BenchMark Ultra IHC/ISH system (Ventana Medical Systems, Inc., Tucson, AZ, USA). Antibody incubation with the respective antibodies was performed according to Table 1: p16 E6H4 (CINtec^®^ mouse monoclonal primary antibody; Ventana Medical Systems, Inc., Tucson, AZ, USA) diluted 1:4, CD4 (CONFIRM anti-CD4 [SP35] rabbit monoclonal primary antibody; Ventana Medical Systems, Inc., Tucson, AZ, USA) diluted 1:4, CD8 (CONFIRM anti-CD8 [SP57] rabbit monoclonal primary antibody; Ventana Medical Systems, Inc., Tucson, AZ, USA) diluted 1:4, FoxP3 (eBioscience [236 A/E7] anti-FOXP3 mouse monoclonal primary antibody; Thermo Fisher Scientific, San Diego, CA, USA) diluted 1:120, and CD1a (Novocastra [MTB1] anti-CD1a mouse monoclonal primary antibody; Leica Biosystems, Newcastle, UK) diluted 1:20. The Dako REAL Envision Detection System, Peroxidase/DAB+, Rabbit/Mouse (Dako, Glostrup, Denmark) was used for detection. Counterstaining was performed with hematoxylin solution. For all TMAs, appropriate hematoxylin and eosin morphological staining were performed as controls.

#### 2.2.2. Assessment of Immunohistochemical Staining

The evaluation of p16, CD4+, CD8+, FoxP3+, and CD1a+ cells were separately performed by an expert pathologist (W.F.) and a trained medical professional (O.A.). The observers were blinded, and controversial cases have been the subject of discussion. For each antibody, the three tissue punches from each patient were evaluated separately. The average of the three punches was then calculated.

For p16, the principle of diffuse block positivity was used, i.e., tumors were classified as p16 positive if there was strong staining of multiple cell layers, and they were classified as p16 negative if the staining intensity was moderate or weak or if the staining was confined to a single cell layer or isolated in stromal cells.

**Figure 1:**
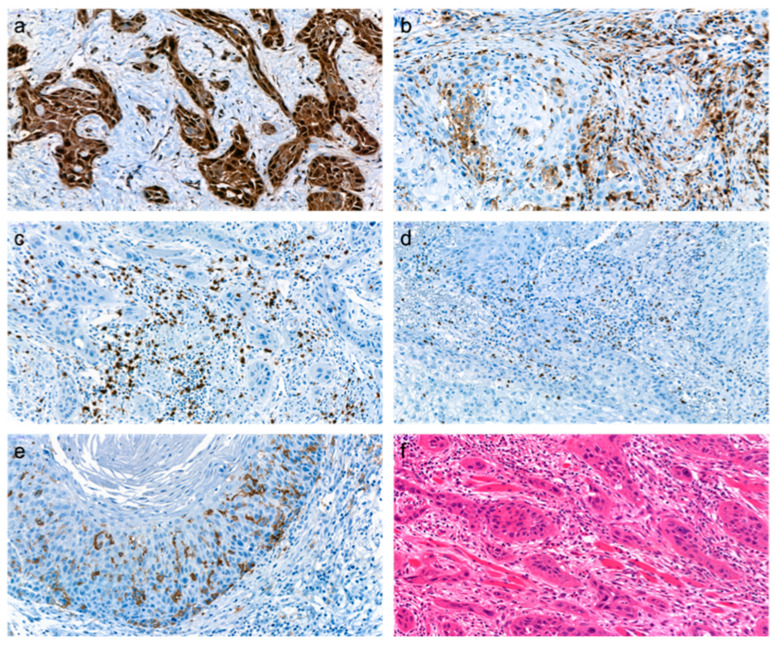
Examples of biopsy specimens from patients with oral squamous cell carcinomas with p16 positivity (**a**), high CD4 expression (**b**), high CD8 expression (**c**), high Forkhead box protein 3 (FoxP3) expression (**d**), high CD1a expression (**e**), and corresponding HE staining (**f**) (magnification 400×).

For CD4, CD8, FoxP3, and CD1a, the absolute number of membrane-stained intratumoral cells were counted in a high-power field (HPF) at 400× magnification, respectively. The HPF describes the area of the tumor where most of the cells of interest are located. The HPF was chosen for the evaluation to achieve the highest possible reproducibility. The evaluation area at 400× magnification corresponded to a rectangle with a height of 256 µm and a width of 432 µm for the microscope used. Counting was performed manually. In addition to the analysis of absolute TIL counts, sums of the average CD4+ and CD8+ cell counts, ratios of the CD4+ and CD8+ cells, as well as ratios of CD8+ and FoxP3+ cells for each patient, were calculated and subsequently evaluated. For CD4, CD8, FoxP3, CD1a, CD4+CD8, CD4/CD8 ratio, and CD8/FoxP3 ratio, a dichotomization into low and high expression groups was performed (Figure 1, Supplementary Figure S1). The subdivision was based on the median value of all tumor samples (low expression ≤ median).

### 2.3. Statistical Analysis

Statistical analysis was performed using IBM SPSS Statistics version 29 (IBM^®^ Deutschland GmbH, Ehningen, Germany). Pearson’s chi-squared test or Fisher’s exact test was used to evaluating associations between clinical data and associations between clinical data and immunohistochemical biomarkers. Univariate survival analyses were performed using the Kaplan–Meier method. The log-rank test was used to compare survival distributions. The Cox proportional hazards model (backward elimination) was used for the multivariate survival analysis. Only relevant variables with *p*-value ≤ 0.100 in the univariate survival analysis were included. Survival analyses were calculated for overall survival (OS) and disease-specific survival (DSS). Overall survival was defined as the time from diagnosis to death from any cause. Disease-specific survival was defined as the time from diagnosis to tumor-related death. All reported p-values are two-sided, and a *p*-value ≤ 0.05 was considered as threshold for statistical significance.

## 3. Results

### 3.1. Patient Characteristics

The study cohort consisted of 131 patients with OSCC. A total of 84 (64.1%) patients were male, and 47 (35.9%) were female. Patient age at diagnosis ranged from 32.91 to 88.32 years, with a median age of 62.57 years. All carcinomas were located in the oral cavity. The exact distribution of locations was as follows: tongue 29 (22.1%), mandible 23 (17.6%), maxilla 9 (6.9), floor of mouth 56 (42.7%), vestibular mucosa 7 (5.3%), soft palate 7 (5.3%). Of the carcinomas of the maxilla and mandible, 18 (56.3%) tumors were located in the area of the edentulous alveolar ridge, 13 (40.6%) tumors were located in the area of the attached gingiva of the still tooth-bearing jaw, and one carcinoma (3.1%) was located peri-implant. All patients were treated by surgery. Adjuvant radiotherapy or chemoradiotherapy was administered to 63 (48.1%) patients. A total of 67 (51.1%) patients were drinkers, and 81 (61.8%) patients reported having smoked. Follow-up after completion of therapy revealed recurrence in 41 (31.3%) cases. The mean follow-up time from diagnosis was 3.61 years (range 0.17–13.34 years).

### 3.2. Associations between Clinicohistopathologic Characteristics and the Use of Smoking and Alcohol Consumption

The results of the analyses of the associations between NSND and clinicohistopathologic features are summarized in Table 2. The analysis revealed that OSCC of NSND was significantly associated with the female gender (*p* < 0.001). Even in isolation, there was a significant association between the female gender and lack of smoking or alcohol consumption (both *p* < 0.001). Further investigations showed that NSND patients were older at the time of diagnosis compared to SD patients (*p* < 0.001). Significant correlations were also found in separate analyses of smokers and drinkers (each *p* < 0.001). A further finding of this study was a significant association between tumor localization and noxious agents. OSCC of NSND occurred significantly more frequently in the maxilla, mandible, and tongue. However, SD tumors showed clustering in the vestibule, the floor of the mouth, and the soft palate (*p* < 0.001). Again, separate analyses for drinking and smoking also confirmed a correlation with the site of tumor development (both *p* < 0.001).

Oral potentially malignant disorders present prior to disease appear to occur significantly more often with NSND (*p* < 0.001). In the separate analysis of NS and ND, this correlation was also observed (*p* < 0.001). Perineural invasion (Pn1) was significantly more common in SD (*p* = 0.039). In the separate analysis of drinkers, the association was even more pronounced (*p* = 0.002).

### 3.3. Associations between NSND, Clinicohistopathologic Characteristics, and Survival

**Figure 2:**
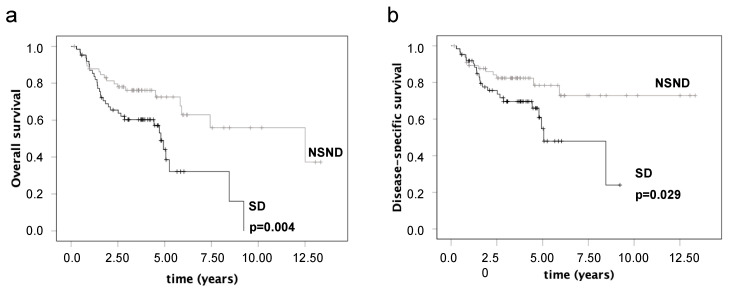
The influence of smoking and drinking on overall survival (**a**) and disease-specific survival (**b**) of patients with oral squamous cell carcinoma.

NSND was significantly associated with better OS (Figure 2, *p* = 0.004) and DSS (Figure 2, *p* = 0.029). Non-smoking alone also showed significantly better OS (75.9% 5-year survival rate of NS vs. 48.8% of smokers, *p* = 0.004) and DSS (82.0% 5-year survival rate of NS vs. 58.7% of smokers, *p* = 0.009). However, the isolated evaluation of alcohol consumption showed only a significantly better OS (70.2% 5-year survival rate of ND vs. 48.3% of drinkers, *p* = 0.042).

Significantly better OS and DSS were also found for the female gender (OS: 74.9% 5-year survival rate of women vs. 51.0% of men, *p* = 0.022; DSS 80.9% 5-year survival rate of women vs. 60.6% of men, *p* = 0.020), N0 stage (OS: 69.2% 5-year survival rate of N0 vs. 42.4% of N+, *p* = 0.004; DSS 75.7% 5-year survival rate of N0 vs. 53.9% of N+, *p* = 0.025), better differentiation (OS: *p* < 0.001; DSS: *p* < 0.001), absence of perineural invasion (OS: 61.6% 5-year survival rate of Pn0 vs. 31.1% of Pn1, *p* = 0.024; DSS 70.3% 5-year survival rate of Pn0 vs. 37.5% of Pn1, *p* = 0.031), L0 stage (OS: 61.9% 5-year survival rate of L0 vs. 41.2% of L1, *p* = 0.014; DSS 70.7% 5-year survival rate of L0 vs. 51.0% of L1, *p* = 0.010) and lower tumor stage (OS: 71.4% 5-year survival rate of UICC stage I/II vs. 50.1% of stage III/IV, *p* = 0.003; DSS 77.6% 5-year survival rate of UICC stage I/II vs. 60.3% of stage III/IV, *p* < 0.032). Presence of oral potentially malignant disorders (OPMDs) (OS: 83.3% 5-year survival rate of presence of OPMD vs. 54.4% of lack of OPMD, *p* = 0.020), higher T stage (51.1% 5-year survival rate of T3/4 vs. 64.2% of T1/2, *p* = 0.003), adjuvant radio- or radiochemotherapy (52.5% 5-year survival rate of adjuvant radio(chemo)therapy vs. 56.4% of lack of adjuvant radio(chemo)therapy, *p* = 0.015), and the presence of recurrence (40.2% 5-year survival rate of recurrence vs. 69.0% of lack of recurrence, *p* = 0.005) were significantly associated with worse OS.

### 3.4. Immunohistochemical Expression and Associations with Clinicohistopathologic Characteristics

In 128 cases, evaluation of p16 expression was possible. Of these, 10 (7.8%) were positive, and 118 (92.2%) were negative. As mentioned above, the remaining immunohistochemical antibodies were divided into high and low expression based on the median. The median values are summarized in Table 3. CD4, CD1a, and FoxP3 evaluation were not possible in one case. The results of the statistical analysis of the immunohistochemical antibodies and the smoking/drinking status are summarized in Table 4.

High CD4+ T-cell infiltration was significantly associated with NSND in the overall group (*p* = 0.008) as well as in separate analyses regarding smoking (*p* = 0.019) and drinking (*p* = 0.005). Similarly, high numbers of CD8+ T cells were shown to be associated with NS (*p* = 0.004) and ND (*p* = 0.003). NSND patients also showed increased CD8+ T cell infiltration (*p* = 0.001). NSND showed significantly greater infiltration with FoxP3+ Tregs (*p* = 0.023). When analyzed separately, NS (*p* = 0.047) and ND (*p* = 0.008) also showed significantly greater infiltration with FoxP3+ cells. Examination of the total number of T cells (CD4+ cells + CD8+ cells) also revealed an association between the increased number of TILs and NSND (*p* = 0.005), NS (*p* = 0.004), and ND (*p* = 0.003). No significant associations were found between p16 and NSND and CD1a and NSND.

### 3.5. Associations of Immunohistochemical Parameters and Survival

**Figure 3:**
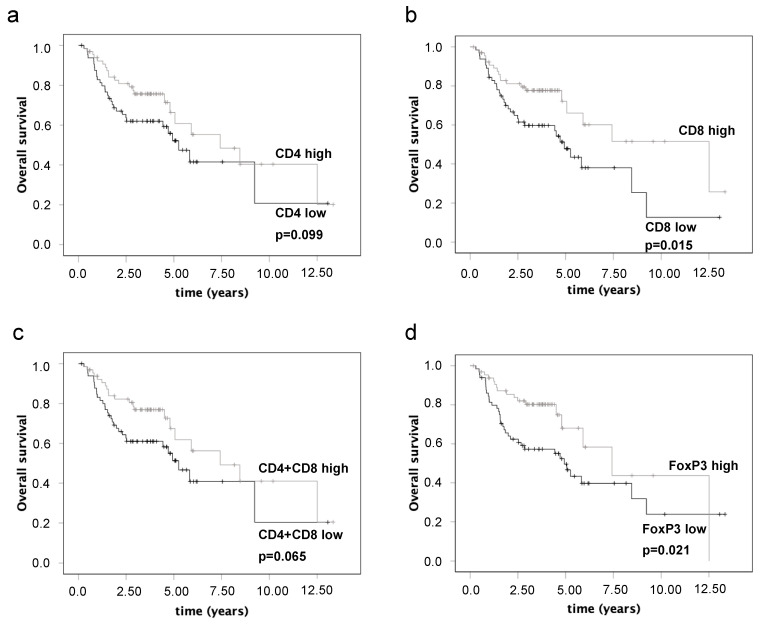
The influence of CD4 (**a**), CD8 (**b**), overall T-cell infiltration (CD4+CD8) (**c**), and Forkhead box protein 3 (FoxP3) (**d**) on overall survival in oral squamous cell carcinoma.

Univariate survival analyses revealed significantly better OS for high CD8+ T-cell infiltration (Figure 3, *p* = 0.015) and high FoxP3+ T-cell infiltration (Figure 3, *p* = 0.021). Increased OS was also shown for high CD4+ T-cell infiltration (Figure 3, *p* = 0.099) and high overall T-cell infiltration (Figure 3, *p* = 0.065), however, without reaching significance. Better DSS was associated with increased infiltration of CD8+ cytotoxic T cells. Again, statistical significance was not reached (77.9% 5-year survival rate of high CD8+ cell infiltration vs. 58.4% of low CD8+ cell infiltration, *p* = 0.07).

### 3.6. Multivariate Survival Analysis

Cox regression analysis showed significantly better OS for NSND (*p* = 0.024), T1/2 (*p* = 0.019), N0 (*p* = 0.009), and lack of relapse (*p* < 0.001) (Table 5).

Multivariate survival analysis for DSS showed that NSND (*p* = 0.027), high CD8+ T-cell tumor infiltration (*p* = 0.031), lack of relapse (*p* < 0.001), UICC stage I/II (*p* = 0.032), and V0 (*p* = 0.035) were predictors of better survival (Table 6).

## 4. Discussion

Smoking and alcohol consumption are considered the major risk factors for the development of OSCC. However, in the last few years, the incidence of patients with OSCC who have never smoked or consumed alcohol is increasingly rising [14,15]. This study investigated differences in clinical and pathological parameters as well as differences in the tumor immune microenvironment by immunohistochemical examination of tumor infiltration of CD4+, CD8+, FoxP3+, and CD1a+ cells in SD and NSND OSCC patients.

OSCC in NSND patients appears to be a distinct subtype of HNSCC that is more common in women and occurs more frequently at different sites of the oral cavity than the typical OSCC of SD [6,14,16]. This present study showed a clustered occurrence of NSND OSCC in the mandible, maxilla, as well as tongue. The classical SD carcinoma, in turn, showed preferential localization in the floor of the mouth, vestibule, and soft palate. This is in line with preliminary studies, which also revealed clustering in the region of the tongue and maxilla [6]. Traditionally, oral cancer is known to be a tumor of men that have a smoking or/and drinking history [6]. Globally, OSCC is more than twice as common among men than women [1]. Additionally, in the current collective, about 2/3 of those affected were men. However, women were significantly more likely to be in the NSND group. Similar observations have been made in other studies so far [6,16,17]. Another interesting finding was that OSCC female patients showed better survival. This result may indicate that OSCC of NSND women is a less aggressive form of tumor.

According to the World Health Organization (WHO) Collaborating Centre for Oral Cancer, oral potentially malignant disorders (OPMDs) are conditions of the oral mucosa that may be associated with an increased risk of developing oral cancer [18,19]. The group of OPMDs includes leukoplakia, erythroplakia, proliferative verrucous leukoplakia, oral submucous fibrosis, oral lichenoid diseases, and actinic keratosis [19]. Interestingly, our studies demonstrated a significantly higher incidence of OPMDs as precursors for OSCC development in the NSND group. The most common OPMD in our study was oral lichen planus or oral lichenoid disorder, which is histologically characterized by a lichenoid subepithelial inflammatory infiltrate of lymphocytes [20]. However, the pathophysiology of OSCC arising from oral lichen planus is rather unknown and requires further investigation [21]. Nevertheless, previous studies that investigated the lichen-caused OSCC have also shown an association with NSND [21]. It is also interesting to note that women are more likely to suffer from oral lichen planus than men [20].

Perineural invasion is a path of cancer spread, which is associated with more aggressive cancer growth [22]. This was also shown in our studies, as the perineural invasion was associated with worse survival. Recent research suggests a mutual attraction of neuronal cells and cancer cells as a cause of perineural invasion, which is largely dependent on neurotrophic factors and their receptors [22]. Interestingly, a dose-dependent release of such neurotrophic factors has been demonstrated in cigarette smoke [22]. Consistent with other studies, we demonstrated an association between the occurrence of perineural invasion and SD, suggesting that OSCC of NSND is a tumor type with less aggressive behavior compared to the group of SD cancer [22,23]. So far, the relationship between the patient’s age at the time of diagnosis and NSND has not been clearly established in previous studies. For example, Li et al. showed in a collective of tongue cancers that NS were younger than smokers at the time of diagnosis [17]. This contrasts with other studies in HNSCC, as well as our results, which show an association of NSND with older age [16]. A further finding of this present study was that NSND OSCC patients had significantly better OS and DSS. As with the findings regarding age at diagnosis, previous data regarding the prognosis of patients with NSND OSCC are somewhat conflicting. In line with our results, a prospective study of 1165 patients with oral cancer showed that NSND patients had significantly better OS and DSS [24]. Similar results were found in other studies [14,21,25]. However, there are also studies that have found no association between NSND and survival. In some cases, NSND has even been associated with a worse prognosis [6,14,26,27]. Consistent with other studies associating a better prognosis with the NSND group, the NSND group in our cohort was older at the time of diagnosis than the SD group. Interestingly, in a study by Bachar et al. showing worse survival in NSND patients, significant results were found only in a subgroup of patients who were less than 40 years old at the time of diagnosis [27]. Therefore, it is reasonable to assume that the following distinct populations of NSND-OCSCC patients may exist [14]: a younger population who may be genetically predisposed and have a more aggressive form of OSCC, and a population of older patients who have a less aggressive form of the tumor [14]. Different pathways of carcinogenesis could be present in the two subgroups.

The human papillomavirus, particularly HPV-16 and HPV-18, has been implicated in oropharyngeal SCC in young NSND patients [6]. However, its role in patients with OSCCs without risk factors such as tobacco or alcohol is not well established [28]. Therefore, to investigate the role of HPV as a possible confounder in our results, we additionally performed p16 staining. In our investigations, no association between NSND OSCC and HPV incidence was found. Furthermore, p16 staining did not correlate with any of the other immunohistochemical markers or clinicopathologic parameters we examined. Therefore, the influence of HPV on the results of this work seems to be largely excluded.

In recent years, the development of immune checkpoint therapies has led to progress in the treatment of HNSCC. However, response to therapy appears to be influenced by the nature of the immunologic milieu of the tumor [29,30]. The immunological changes caused by noxious agents such as smoking and alcohol consumption are diverse and complex but also suggest differences in the tumor immune microenvironment between NSND and SD OSCC [5,7]. Better and more individualized therapeutic approaches for the subset of NSND OSCC may be developed with more detailed knowledge of these differences. The results of this current study demonstrated an association between NSND and an increased number of TILs. This association was shown both in the whole T cell population as well as individually for the groups of CD8+ cytotoxic T cells, CD4+ T cells, and here, also for the subset of FoxP3+ Tregs. Similar results were found in a study by Foy et al. in HPV-negative OSCC patients that showed an association between NSND, total T-cell tumor infiltration, and CD4+, as well as CD8+ positive TILs [5]. Interestingly, contradictory results were found in other tumor entities. For example, in non-small cell lung cancer, high expression of FoxP3 was associated with smoking [31]. One reason for these conflicting results may be the influence of additional alcohol consumption [5]. Alcohol forms a recognized risk factor for OSCC, but not for lung cancer [3,5]. Tregs are involved in maintaining immunological tolerance to host tissues and are therefore considered suppressors of the antitumor immune response [32,33]. Although these characteristics suggest a worse outcome, high FoxP3+ TILs were associated with a better prognosis in our studies. Similar results were found in a former meta-analysis [33]. Several reasons have been discussed as to why FoxP3+ cells are associated with a better outcome despite known antitumor immune activity. One reason for this may be that a high expression of FoxP3+ TILs reflects an overall higher T cell infiltration, and thus, the cytotoxic beneficial effects of CD8+ T cells outweigh the Treg-associated antitumor immune activities [33,34]. CD8+ T cells have the ability to directly attack and destroy tumor cells by binding to MHC class I molecules [33]. Several former studies showed that high CD8+ TIL expression was associated with better survival [5,33,35]. This current study also associated high CD8+ cell infiltration with better survival in multivariate analysis, in addition to NSND, freedom from recurrence, lower tumor stage, and lack of vascular infiltration. A further group of immune cells we investigated in this study were DCs. Preliminary studies have demonstrated decreased expressions of immature and mature DCs in OSCC in smokers compared with NS [36]. DCs are antigen-presenting cells (APCs) characterized by their unique ability to stimulate naive T cells, initiate primary immune responses, and participate in the induction of central and peripheral immunological tolerance [37]. This current study did not reveal any significant association between NSND and CD1a expression. On the one hand, tumor mutational burden (TMB) has been shown to be a predictive factor for response to immunotherapy. Further smoking is associated with increased mutational burden [2,38]. On the other hand, our findings conclude OSCC of NSND patients is more immunogenic shown by higher expression of TILs. The outcome of immune checkpoint therapy has been associated with the quality and quantity of immune cells within the tumor environment [29,39]. Therefore, patients with NSND-OSCC may be a group of patients who could benefit most from immune checkpoint therapy. More studies are needed to test this hypothesis.

## 5. Conclusions

In conclusion, NSND OSCC seems to be a distinct type of OSCC as it appears to be less aggressive and shows a better outcome. NSND OSCC has unique clinical and pathologic characteristics, as evidenced by the fact that it is a tumor that predominantly affects women and is associated with a better outcome. In addition, NSND OSCC has a more immunogenic microenvironment, as reflected by higher TIL expression. In summary, the results of this study may help to develop specific treatment solutions for this relevant group of OSCC patients.

## Figures and Tables

**Table 1:** Antibody characteristics.

Antibody	Dilution	Heat Retrieval	Cell Conditioner	Incubation Time [min]
p16	1:4	yes	CC1	60
CD4	1:4	yes	CC1	44
CD8	1:4	yes	CC1	32
CD1a	1:20	yes	CC1	28
FoxP3	1:120	yes	CC1	32

**Table 2:** Associations between NSND and clinicohistopathological characteristics.

Noxious	Smoking				Drinking				Smoking and Drinking			
	No	Yes	N	*p*-Value	No	Yes	N	*p*-Value	No	Yes	N	*p*-Value
sex												
male	20 (40.0%)	64 (79.0%)	84 (64.1%)		26 (41.9%)	57 (85.1%)	83 (64.3%)		30 (44.8%)	53(85.5%)	83 (64.3%)	
female	30 (60.0%)	17 (21.0%)	47 (35.9%)	<0.001	36 (58.1%)	10 (14.9%)	46 (35.7%)	<0.001	37 (55.2%)	9 (14.5%)	46 (35.7%)	<0.001
Age at diagnosis												
<71 years	27 (54.0%)	71 (87.7%)	98 (74.8%)		37 (59.7%)	59 (88.1%)	96 (74.4%)		40 (59.7%)	56 (90.3%)	96 (74.4%)	
≥71 years	23 (46.0%)	10 (12.3%)	33 (25.2%)	<0.001	25 (40.3%)	8 (11.9%)	33 (25.6%)	<0.001	27 (40.3%)	6 (9.7%)	33 (25.6%)	<0.001
Localization												
tongue	12 (24.0%)	17 (21.0%)	29 (22.1%)		19 (30,6%)	10 (14.9%)	29 (22.5%)		20 (29.9%)	9 (14.5%)	29 (22.5%)	
mandible	12 (24.0%)	11 (13.6%)	23 (17.6%)		14 (22.6%)	7 (10.4%)	21 (16.3%)		14 (20.9%)	7 (11.3%)	21 (16.3%)	
maxilla	9 (18.0%)	0 (0.0%)	9 (6.9%)		9 (14.5%)	0 (0.0%)	9 (7.0%)		9 (13.4%)	0 (0.0%)	9 (7.0%)	
floor of mouth	12 (24.0%)	44 (54.3%)	56 (42.7%)		16 (25.8%)	40 (59.7%)	56 (43.4%)		19 (28.4%)	37 (59.7%)	56 (43.4%)	
vestibule	3 (6.0%)	4 (4.9%)	7 (5.3%)		2 (3.2%)	5 (7.5%)	7 (5.4%)		3 (4.5%)	4 (6.5%)	7 (5.4%)	
soft palate	2 (4.0%)	5 (6.2%)	7 (5.3%)	<0.001	2 (3.2%)	5 (7.5%)	7 (5.4%)	<0.001	2 (3.0%)	5 (8.1%)	7 (5.4%)	<0.001
T-stage												
T1-2	28 (56.0%)	52 (64.2%)	80 (61.1%)		38 (61.3%)	41 (61.2%)	79 (61.2%)		40 (59.7%)	39 (62.9%)	79 (61.2%)	
T3-4	22 (44.0%)	29 (35.8%)	51 (38.9%)	0.363	24 (38.7%)	26 (38.8%)	50 (38.8%)	1.000	27 (40.3%)	23 (37.1%)	50 (38.8%)	0.722
N-Stage												
N0	30 (61.2%)	51 (63.0%)	81 (62.3%)		37 (60.7%)	42 (62.7%)	79 (61.7%)		39 (59.1%)	40 (64.5%)	79 (61.7%)	
N+	19 (38.8%)	30 (37.0%)	49 (37.7%)	0.854	24 (39.3%)	25 (37.3%)	49 (38.3%)	0.857	27 (40.9%)	22 (35.5%)	49 (38.3%)	0.587
M-stage												
M0	50 (100.0%)	80 (100.0%)	130 (100.0%)		62 (100.0%)	66 (100.0%)	128 (100.0%)		67 (100.0%)	61 (100.0%)	128 (100.0%)	
M1	0 (0.0%)	0 (0.0%)	0 (0.0%)		0 (0.0%)	0 (0.0%)	0 (0.0%)		0 (0.0%)	0 (0.0%)	0 (0.0%)	
G-Stage												
G1-2	43 (86.0%)	63 (77.8%)	106 0,809%		52 (83.9%)	52 (77.6%)	104 (80.6%)		56 (83.6%)	48 (77.4%)	104 (80.6%)	
G3-4	7 (14.0%)	18 (22.2%)	25 0(19.1%)	0.360	10 (16.1%)	15 (22.4%)	25 (19.4%)	0.384	11 (16.4%)	14 (22.6%)	25 (19.4%)	0.504
Pn-Stage												
Pn0	48 (96.0%)	70 (86.4%)	118 (90.1%)		61 (98.4%)	55 (82.1%)	116 (89.9%)		64 (95.5%)	52 (83.9%)	116 (89.9%)	
Pn1	2 (4.0%)	11 (13.6%)	13 (9.9%)	0.130	1 (1.6%)	12 (17.9%)	13 (10.1%)	0.002	3 (4.5%)	10 (16.1%)	13 (10.1%)	0.039
L-Stage												
L0	46 (92.0%)	68 (84.%)	114 (87.0%)		57 (91.9%)	55 (82.1%)	112 (86.8%)		61 (91.%)	51 (82.3%)	112 (86.8%)	
L1	4 (8.0%)	13 (16.0%)	17 (13.0%)	0.284	5 (8.1%)	12 (17.9%)	17 (13.2%)	0.122	6 (9.0%)	11 (17.7%)	17 (13.2%)	0.193
V-Stage												
V0	49 (98.0%)	79 (97.5%)	128 (97.7%)		62 (100.0%)	64 (95.5%)	126 (97.7%)		66 (98.5%)	60 (96.8%)	126 (97.7%)	
V1	1 (2.0%)	2 (2.5%)	3 (2.3%)	1.000	0 (0.0%)	3 (4.5%)	3 (2.3%)	0.245	1 (1.5%)	2 (3.2%)	3 (2.3%)	0.608
Lymphoma												
no	47 (94.0%)	80 (98.8%)	127 (96.9%)		58 (93.5%)	67 (100.0%)	125 (96.9%)		63 (94.0%)	62 (100.0%)	125 (96.9%)	
yes	3 (6.0%)	1 (12.0%)	4 (3.1%)	0.155	4 (6.5%)	0 (0.0%)	4 (3.1%)	0.051	4 (6.0%)	0 (0.00%)	4 (3.1%)	0.120
OPMD												
no	33 (66.0%)	80 (98.8%)	113 (86.3%)		47 (75.8%)	64 (95.5%)	111 (86.0%)		50 (74.6%)	61 (98.4%)	111 (86.0%)	
yes	17 (34.0%)	1 (1.2%)	18 (13.7%)	<0.001	15 (24.2%)	3 (4.5%)	18 (1.4%)	<0.001	17 (25.4%)	1 (1.6%)	18 (1.4%)	<0.001

**Table 3:** Median-value of intratumoral stained cells.

Antibody	CD4	CD8	CD4+CD8	FoxP3	CD1a	CD8/FoxP3	CD4/CD8
N	130	131	130	130	130	128	130
Median	201.83	72.33	285.00	41.50	14.33	1.59	2.80
Std. Deviation	155.37	85.21	224.56	33.65	15.60	2.95	4.64
Minimum	11.67	1.67	13.33	0.00	0.33	0.14	0.55
Maximum	737.67	423.00	1047.50	170.33	92.00	29.54	41.00

**Table 4:** Relationship between NSND and p16, CD4, CD8, sum of CD4 and CD8, FoxP3, CD1a, CD4/CD8 ratio, CD8/FoxP3 ratio.

Noxious	Smoking				Drinking				Smoking and Drinking			
	No	Yes	N	*p*-Value	No	Yes	N	*p*-Value	No	Yes	N	*p*-Value
p16												
negative	44 (89.8%)	74 (93.7%)	118 (92.2%)		54 (91.5%)	62 (92.5%)	116 (92.1%)		58 (90.6%)	58 (93.5%)	116 (92.1%)	
positive	5 (10.2%)	5 (6.3%)	10 (7.8%)	0.505	5 (8.5%)	5 (7.5%)	10 (7.9%)	1.000	6 (9.4%)	4 (6.5%)	10 (7.9%)	0.744
CD4												
low	18 (36.0%)	47 (58.0%)	65 (49.6%)		23 (37.1%)	42 (62.7%)	65 (50.4%)		26 (38.8%)	39 (62.9%)	65 (50.4%)	
high	32 (64.0%)	34 (42.0%)	66 (50.4%)	0.019	39 (62.9%)	25 (37.3%)	64 (49.6%)	0.005	41 (61.2%)	23 (37.1%)	64 (49.6%)	0.008
CD8												
low	16 (32.0%)	48 (59.3%)	64 (48.9%)		22 (35.5%)	42 (62.7%)	64 (49.6%)		24 (35.8%)	40 (64.5%)	64 (49.6%)	
high	34 (68.0%)	33 (40.7%)	67 (51.1%)	0.004	40 (64.5%)	25 (37.3%)	65 (50.4%)	0.003	43 (64.2%)	22 (35.5%)	65 (50.4%)	0.001
CD4+CD8												
low	17 (34.0%)	49 (61.3%)	66 (50.8%)		23 (37.7%)	43 (64.2%)	66 (51.6%)		26 (39.4%)	40 (64.5%)	66 (51.6%)	
high	33 (66.0%)	31 (38.8%)	64 (49.2%)	0.004	38 (62.3%)	24 (35.8%)	62 (48.4%)	0.004	40 (60.6%)	22 (35.5%)	62 (48.4%)	0.005
FoxP3												
low	19 (38.0%)	46 (57.5%)	65 (50.0%)		23 (37.7%)	42 (62.7%)	65 (50.8%)		27 (41.5%)	38 (58.5%)	65 (50.8%)	
high	31 (62.0%)	34 (42.5%)	65 (50.0%)	0.047	38 (62.3%)	25 (37.3%)	63 (49.2%)	0.008	39 (59,1%)	24 (38.7%)	63 (49.2%)	0.023
CD1a												
low	23 (46.0%)	45 (56.3%)	68 (52.3%)		30 (49.2%)	37 (55.2%)	67 (52.3%)		31 (47.0%)	36 (58.1%)	67 (52.3%)	
high	27 (54.0%)	35 (43.8%)	62 (47.7%)	0.283	31 (50.8%)	30 (44.8%)	61 (47.7%)	0.595	35 (53.0%)	26 (41.9%)	61 (47.7%)	0.221
CD4/CD8												
low	29 (58.0%)	36 (45.0%)	65 (50.0%)		30 (49.2%)	33 (49.3%)	63 (49.2%)		34 (51.5%)	29 (46.8%)	63 (49.2%)	
high	21 (42.0%)	44 (55.0%)	65 (50.0%)	0.207	31 (50.8%)	34 (50.7%)	65 (50.8%)	1.000	32 (48.5%)	33 (53.2%)	65 (50.8%)	0.601
CD8/FoxP3												
low	20 (40.8%)	44 (55.7%)	64 (50.0%)		29 (49.2%)	34 (50.7%)	63 (50.0%)		30 (46.9%)	33 (53.2%)	63 (50.0%)	
high	29 (59.2%)	35 (44.3%)	64 (50.0%)	0.145	30 (50.8%)	33 (49.3%)	63 (50.0%)	1.000	34 (53.1%)	29 (46.8%)	63 (50.0%)	0.593

**Table 5:** Univariate and multivariate analysis (backward elimination): Overall survival (encountered *p*-values).

Parameter (*n* = 131)	Univariate (Log-Rank)	Multivariate (Cox Regression)	
	*p*-Value	*p*-Value (Step 11)	HR ^a^ (95% CI ^b^)
Noxious (NSND vs. SD)	0.004	0.024	2.071 (1.099–3.903)
T-stage (T1/2 vs. T3/4)	0.003	0.019	2.055 (1.127–3.749)
N-stage (N0 vs. N+)	0.004	0.009	2.180 (1.217–3.903)
G-stage (G1 vs. G2 vs. G3 vs. G4)	<0.001		
L-stage (L0 vs. L1)	0.014		
V-stage (V0 vs. V1)	0.057	0.056	4.330 (0.962–19.482)
Pn-stage (Pn0 vs. Pn1)	0.024		
Gender (female vs. male)	0.022		
UICC-stage (I/II vs. III/IV)	0.003		
Relapse (no relapse vs. relapse)	0.005	<0.001	2.999 (1.661–5.417)
OPMD (no OPMD vs. OPMD)	0.020	0.112	0.380 (0.115–1.254)
Adj. RCT (no RCT vs. RCT)	0.015		
CD4 (low expression vs. high expression)	0.099		
CD8 (low expression vs. high expression)	0.015	0.074	0.551 (0.287–1.058)
FoxP3 (low expression vs. high expression)	0.021		

^a^ hazard ratio; ^b^ confidence interval; adjuvant radio(chemo)therapy = adj. RCT.

**Table 6:** Univariate and multivariate analysis (backward elimination): Disease-specific survival (encountered *p*-values).

Parameter (*n* = 131)	Univariate (Log-Rank)	Multivariate (Cox Regression)	
	*p*-Value	*p*-Value (Step 7)	HR ^a^ (95% CI ^b^)
Noxious (NSND vs. SD)	0.029	0.027	2.326 (1.099–4.924)
T-stage (T1/2 vs. T3/4)	0.062		
N-stage (N0 vs. N+)	0.025		
G-stage (G1 vs. G2 vs. G3 vs. G4)	<0.001		
L-stage (L0 vs. L1)	0.010		
V-stage (V0 vs. V1)	0.016	0.035	5.994 (1.135–31.660)
Pn-stage (Pn0 vs. Pn1)	0.031	0.056	2.884 (0.971–8.565)
Gender (female vs. male)	0.020		
UICC-stage (I/II vs. III/IV)	0.032	0.032	2.335 (1.074–5.075)
Relapse (no relapse vs. relapse)	<0.001	<0.001	5.773 (2.587–12.881)
OPMD (no OPMD vs. OPMD)	0.061		
CD8 (low expression vs. high expression)	0.07	0.031	0.426 (0.196–0.924)

^a^ hazard ratio; ^b^ confidence interval.

## Data Availability

Data can be obtained by industry scientists who conducted independent work upon request. Data are not stored on publicly available servers.

## Supplementary Materials

The following supporting information can be downloaded at: https://www.mdpi.com/article/10.3390/cancers15102688/s1, Supplementary Figure S1: Negative controls of immunohistochemical stainings.
