# Supplementary figures and images for: OSCC in Never-Smokers and Never-Drinkers Is Associated with Increased Expression of Tumor-Infiltrating Lymphocytes and Better Survival
Source: Cancers (Basel). 2023 May 10;15(10):2688. doi: 10.3390/cancers15102688 (PMC10216056; doi:10.3390/cancers15102688)

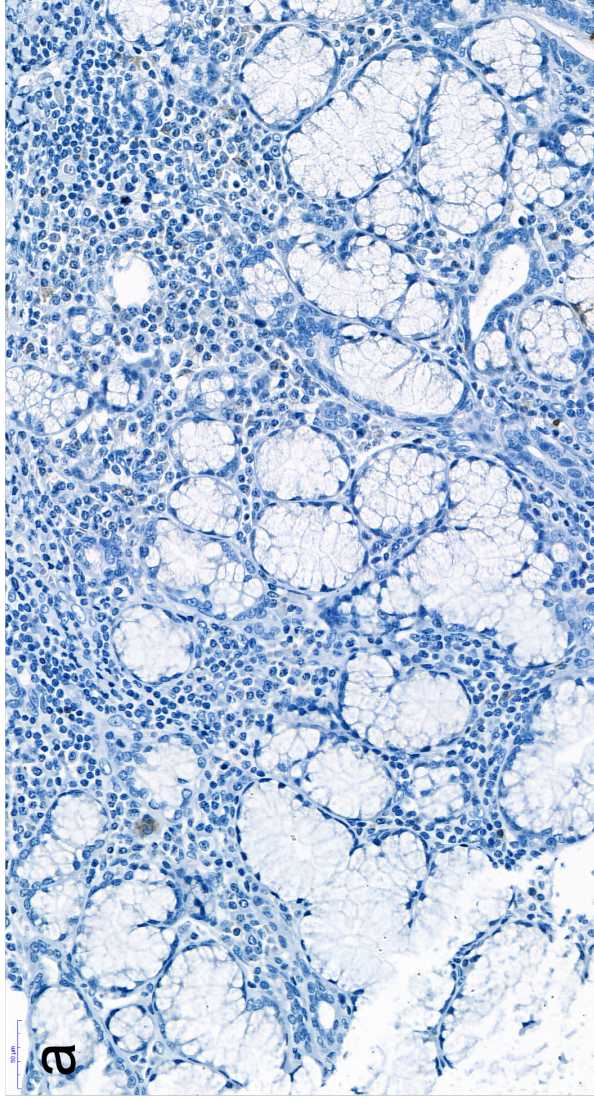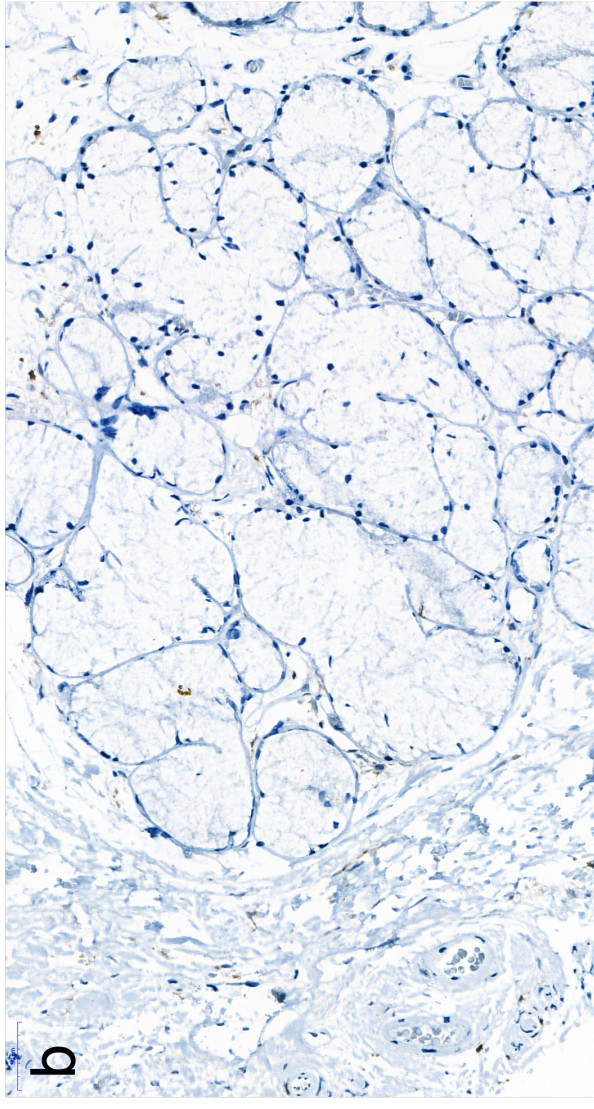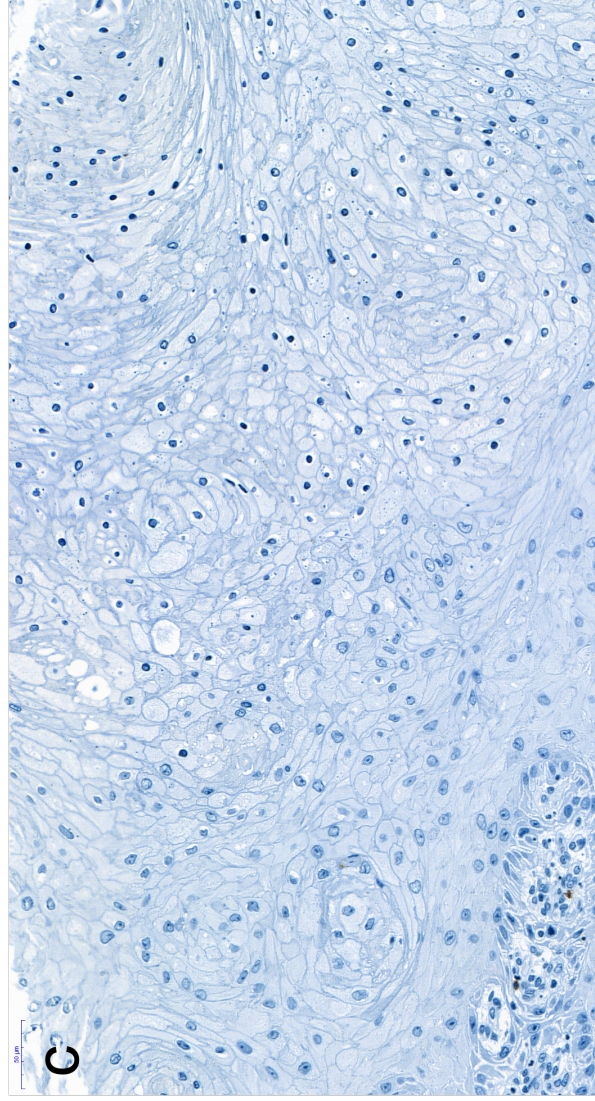

Supplement: Supplementary file 1 [file cancers-15-02688-s001.zip › cancers-2362547-supplementary.pdf]
